# Adenosine A_2A_ receptor in schizophrenia: an in vivo brain PET imaging study

**DOI:** 10.1007/s00213-021-05900-0

**Published:** 2021-06-26

**Authors:** Tiago Reis Marques, Sridhar Natesan, Eugenii A. Rabiner, Graham E. Searle, Roger Gunn, Oliver D. Howes, Shitij Kapur

**Affiliations:** 1 Psychiatric Imaging Group, MRC London Institute of Medical Sciences (LMS), Hammersmith Hospital, Imperial College London, London, UK; 2 Psychiatric Imaging Group, Institute of Clinical Sciences (ICS), Faculty of Medicine, Imperial College London, London, UK; 3 Department of Psychosis Studies, Institute of Psychiatry, Psychology and Neuroscience, Kings College London, London, UK; 4 Centre for Neuroimaging Sciences, Institute of Psychiatry, King’s College London, London, UK; 5 Centre for Imaging Sciences, London, UK

**Keywords:** A2A, Schizophrenia, PET, Imaging, [^11^C]SCH442416, Antipsychotics

## Abstract

Adenosine A_2A_ receptors are highly enriched in the basal ganglia system, a region that is functionally implicated in schizophrenia. Preclinical evidence suggests a cross-regulation between adenosine A_2A_ and dopamine D_2_ receptors in this region and that it is linked to the sensitization of the dopamine system. However, the relationship between A_2A_ receptor availability and schizophrenia has not been directly examined in vivo in patients with this disorder. To investigate, using positron emission tomography (PET), the availability of A_2A_ receptors in patients diagnosed with schizophrenia in comparison to matched healthy controls. A_2A_ receptor availability was measured using the PET tracer [^11^C]SCH442416. Twelve male patients with chronic schizophrenia were compared to 13 matched healthy subjects. All patients were medicated with antipsychotics and none presented with any motor or extrapyramidal symptoms. Binding potential (BP_ND_), a ratio measure between specific and non-specific tracer uptake, were compared between the groups for the caudate, putamen, accumbens and globus pallidum. There was no differences between A_2A_ receptor binding potential (BP_ND_) of schizophrenia patients in the caudate (*p* = 0.16), putamen (*p* = 0.86), accumbens (*p* = 0.44) and globus pallidum (*p* = 0.09) to that of matched healthy subjects. There was also no significant correlation between [11C]SCH442416 binding and severity of psychotic symptoms (*p* = 0.2 to 0.82) or antipsychotic dosage (*p* = 0.13 to 0.34). By showing that A2A receptor availability in medicated patients with chronic male schizophrenia is not different than in healthy controls, this study does not support the primary role of this receptor in the pathogenesis of schizophrenia.

## Introduction

Schizophrenia is a chronic mental health disorder that affects approximately 1% of the population. Although the precise neurobiological mechanisms underlying schizophrenia are still unknown, several lines of evidence show that striatal abnormalities are a core component of the pathophysiology of this disorder (Howes and Kapur 2009). Adenosine regulates dopaminergic neurotransmission, making it of potential relevance to understanding the striatal dopaminergic abnormalities seen in the illness. In the striatum, it acts on adenosine 2A receptors (A2AR) that are expressed on the bodies of indirect motor pathway medium spiny neurons (Svenningsson et al. 1999). These striatopallidal neurons also express dopamine D2 receptors (D2Rs). The modulatory role of dopaminergic function by adenosine is related to the ability of A2A receptors to create intermolecular interactions between A2AR and D2R, with the formation of receptor heteromers. By signalling via opposite G-protein coupling mechanisms, the activation of A2AR inhibits the dopaminergic function (Kull et al. 1999; Tozzi et al. 2011). This points to a cross-regulation between the two systems and suggests that A2AR activation can modulate dopaminergic signalling (Fredduzzi et al. 2002; Soria et al. 2006; Rial et al. 2004). Therefore, it has been suggested that A2AR could have potential implications in the pathogenesis of schizophrenia (Boison et al. 2012).

According to the adenosine hypothesis of schizophrenia, reduced adenosinergic activity would trigger a compensatory upregulation of striatal A2AR, which in turn would suppress the activation of the indirect pathway inducing a hyperdopaminergic state (Lara and Souza 2000; Lara et al. 2006; Boison et al. 2012). Preclinical studies have shown that genetically engineered mice with a reduced expression of A2A receptors present an attenuated locomotor responses to amphetamine and cocaine (Bastia et al. 2005; Filip et al. 2006; Soria et al. 2006). Interestingly, pharmacological evidence has supported the role of adenosine modulators in the treatment of schizophrenia. Allopurinol, a drug that acts as a xanthine oxidase inhibitor and therefore increases adenosine levels, has been shown to be beneficial as an add-on treatment to antipsychotics in reducing mostly positive symptoms (Lara et al. 2001; Brunstein et al. 2005). A recent meta-analysis of six trials in schizophrenia has shown that adenosine modulators are superior to placebo in reducing total PANSS scores in schizophrenia patients (SMD =  − 1.07) (Hirota and Kishi 2013).

However, very few studies have investigated A2A receptors in patients with schizophrenia. Two post-mortem studies in schizophrenia both showed a significant increase (35 to 55%) in the A2A receptor (A2AR) in the basal ganglia of patients with this disorder when compared to healthy controls (Kurumaji and Toru 1998; Deckert et al. 2003). A more recent post-mortem study has however showed a reduction in A2AR binding as well as a reduction in A2AR protein and mRNA levels (Villar-Menéndez et al. 2014). However, to our knowledge, there has been no in vivo studies have in patients with schizophrenia. In this study, we have used PET imaging to assess for the first-time A2AR availability in male patients with schizophrenia using the [^11^C]SCH442416 tracer. We hypothesised that patients with schizophrenia have an upregulation of A2AR in the striata when compared to healthy controls and that greater A2AR availability will be directly associated with symptom severity.

## Methods

### Participants

Patients with schizophrenia (*n* = 12) were recruited from the South London and Maudsley Foundation NHS Trust, and were compared to matched healthy controls (*n* = 13). All participants gave written informed consent to participate after receiving a full description of the study. The study protocol was approved by the National Research Ethics Service (NRES) and permission to administer radioactive substances was granted by the Administration of Radioactive Substances Advisory Committee (ARSAC), UK. All subjects had a physical, psychiatric and neurological examination. Diagnosis was performed by reviewing patients medical notes and the use of the Structured Clinical Interview for DSM-5 (SCID) (First et al. 2015). Symptom severity was assessed using the Positive and Negative Syndrome Scale (PANSS) (Kay et al. 1987). Inclusion criteria for patients were a DSM-5 diagnosis for schizophrenia or schizophreniform disorder, at least one rating of moderate severity (PANSS ≥ 4) on the PANSS positive scale, clinically stable in a non-acute phase for at least 4 weeks prior to inclusion, and treatment with a stable dose of a second-generation antipsychotic (except clozapine) for at least 12 weeks. Inclusion criteria for healthy controls were absence of any DSM-5 diagnosis or any psychotic illness in first-degree relatives. Exclusion criteria for all groups included significant medical illnesses, history of head trauma or injury with loss of consciousness longer than 1 h; pregnancy, organic psychosis; learning disabilities or lack of English fluency, treatment with clozapine, consuming over 500 mg per day of caffeine, smoking more than 20 cigarettes per day, history of substance abuse or dependence or other neurological or psychiatric disorders other than schizophrenia in the patient group. All patients had a period of abstinence of caffeine of at least 24 h. This interval was calculated based on the mean half-life of caffeine in plasma, which is approximately 5 h (Brachtel and Richter 1992). Drug screens were done on the day of the scan to exclude the current use of psychoactive drugs. Clinical measures were recorded at the PET scan, including physical examination, urine drug screen and medication history. Antipsychotic doses were converted to chlorpromazine equivalents (Atkins et al. 1997; Woods 2003). Handedness was assessed using the Annett Hand Preference Questionnaire (Annett 1970).

### Neuroimaging evaluation

#### Image acquisition

PET and MR imaging was performed at Imanova Ltd, London, UK. Participants were instructed to refrain from caffeine, tobacco and alcohol for at least 12 h before scanning. MRI scans were acquired with a 32-channel head coil on a Siemens Magnetom Verio, 3-T MRI scanner and included a T1-weighted magnetization prepared rapid gradient echo sequence (MPRAGE; time repetition (TR) = 2300 ms, time echo (TE) = 2.98 ms, flip angle of 9°, time to inversion (TI) = 900 ms, matrix = 240 × 256) for co-registration with the PET images; fast GM T1 inversion recovery (FGATIR; TR = 3000 ms, TE = 2.96 ms, flip angle of 8°, TI = 409 ms, matrix = 240 × 256) (REF) and fluid and WM suppression (FLAWS; TR = 5000 ms, TE = 2.94 ms, flip angle of 5°, TI = 409/1100 ms, matrix = 240 × 256) sequences for improving delineation of subcortical brain regions. All sequences used a 1-mm^3^ voxel size, anteroposterior phase encoding direction and a symmetric echo.

PET scans were acquired on a Siemens Biograph Hi-Rez 6 PET-CT scanner (Erlangen, Germany) following the injection of an intravenous bolus of [^11^C]SCH442416. Subjects were positioned supine with their transaxial planes parallel to the line intersecting the anterior–posterior commissure line. Head position was maintained with the help of individualised foam holders, monitored by video and subjects were repositioned if movement was detected. Subjects were in a resting state with low light. Intrascan notes for participant’s movement were acquired during scanning. Dynamic emission data were acquired continuously for 90 min following the injection of [^11^C]SCH442416. The dynamic images were reconstructed into 26 frames (8 × 15 s, 3 × 60 s, 5 × 120 s, 5 × 300 s and 5 × 600 s), using a filtered back projection algorithm (direct inversion Fourier transform) with a 128 × 128 matrix, zoom of 2.6, producing images with isotropic voxel size of 2 × 2 × 2 mm^3^ and a 5-mm isotropic Gaussian filter. Corrections were applied for attenuation, randoms and scatter. Arterial plasma samples were collected during the scan to enable metabolite corrected input functions to be determined.

##### Data analysis

Data were analysed using Imanova’s in-house MIAKAT™ software package (www.miakat.org). MIAKAT™ implements a robust and consistent analysis pipeline with built-in audit trail and pre-specified QC points, whereby the analyst is required to inspect results of intermediate stages (for example: checking the success of automated brain extraction or assessing the quality of model fits). The specifics of the analyses used are described in the following sections.

##### Image processing and definition of regions of interest

Each subject’s structural MRI image underwent brain extraction, grey matter segmentation and was co-registered to a standard reference space (MNI152) (Grabner et al. 2006). The MNI152 template brain image and associated atlas (CIC atlas) (Tziortzi et al. 2011) were nonlinearly warped to the subject’s MR image to enable automated definition of regions of interest (ROIs). The ROIs defined in this manner were dorsal caudate, putamen, accumbens, globus pallidus and cerebellum. Dynamic PET images were registered to each subject’s MRI scan and corrected for subject motion using a frame-to-frame registration process with a normalised mutual information cost function. ROIs were applied to the dynamic PET data to derive regional time-activity curves (TACs).

##### Kinetic modelling

11C]SCH442416 is a radioligand with limited data available in the published literature to inform on the optimal techniques for the quantification of A2A receptor availability (Brooks et al. 2010; Ramlackhansingh et al. 2011). [^11^C]SCH442416 presents an atypical and somewhat inconsistent tissue time-activity curve (TAC) profile that has not yet been fully explained. In particular, a slow accumulative component to the tissue signal is seen in many scans, which is not well-described by standard PET kinetic models. Previous investigators (Hinz et al. 2003; Brooks et al. 2010) have sought to explicitly account for this component by fitting a plasma-input model that partitions the signal into a reversible component which is assumed to correspond to interaction with the A2A receptors and a slow, irreversible component of unknown origin that becomes evident later in the scan. However, this approach did not fit our data well and produced highly variable results, and hence was not used in further analysis here. Instead, an alternative approach was used which assumes that (a) the contribution of this irreversible component to the overall tissue is negligible during the first 15 min of scan data, and (b) BP_ND_ can be estimated adequately by fitting the simplified reference tissue compartmental model (SRTM) to just the first 15 min of scan data. The SRTM was implemented using a basis function approach (Gunn et al. 1997), and used the cerebellum as reference region.

### Statistical analysis

Statistical analysis was performed with SPSS (version 23) for MAC OS X. All data are presented as mean ± SD, and the level *α* was set for all comparisons at *P* < 0.05. As this is an exploratory study, *P* values were not adjusted for multiple comparisons. For all variables, variance homogeneity and normal distribution were assessed with Kolmogorov–Smirnov tests. Since PET and clinical data were normally distributed we proceeded with parametric tests. Based on the post-mortem data and preclinical literature, the primary regions of interest were the caudate and putamen. In secondary analyses, we investigated group differences in other basal ganglia regions. To determine whether there was an effect of group on BP_ND_ values and on socio-demographic and clinical data, independent *t* tests were performed as appropriate. We interrogated correlations between PET and clinical data using Pearson’s *r*.

## Results

### Demographic and clinical characteristics

Twelve male individuals with schizophrenia and 13 male healthy control subjects were studied. The demographic and clinical characteristics of the subjects are presented in Table 1 along with information on the injected tracer. No significant demographic differences between the patient and healthy controls group were observed. Symptoms were evaluated using the PANSS (mean PANSS total score = 61.5, SD: ± 9.70). All patients were on an antipsychotic at the time of the scan and antipsychotic blood levels showed values within the expected range for the dose (Taylor et al. 2018).

**Table 1:** Demographic, clinical and tracer dose characteristics of schizophrenia patients and healthy controls groups

	Patients (*n* = 12)		Healthy controls (*n* = 13)		Test statistic	
	Mean	SD	Mean	SD	*F* (df)	*p*
Age at scan (mean years, SD)	38.5	9.68	34.2	9.08	0.112 (23)	0.25
Gender (male/female)	12/00		13/00			
Handedness: right-handed (%)	100%		100%			
PANSS total score, baseline (mean, SD)	61.5	9.70	n/a	n/a	n/a	n/a
Duration of illness in months (mean, SD)	149.5	78.3	n/a	n/a	n/a	n/a
Total antipsychotic dose at baseline (mean CPZ equivalents, SD)	360.3	274.0	n/a	n/a	n/a	n/a
Antipsychotics used (% of second generation)	92%		n/a	n/a	n/a	n/a
Injected activity (MBq)	419.3	42.7	381.2	76.2	0.843 (23)	0.14
Injected mass (μg/Kg)	3.35	1.35	3.12	1.06	0.354 (23)	0.63

### A2A expression in the striatum

**Fig. 1:**
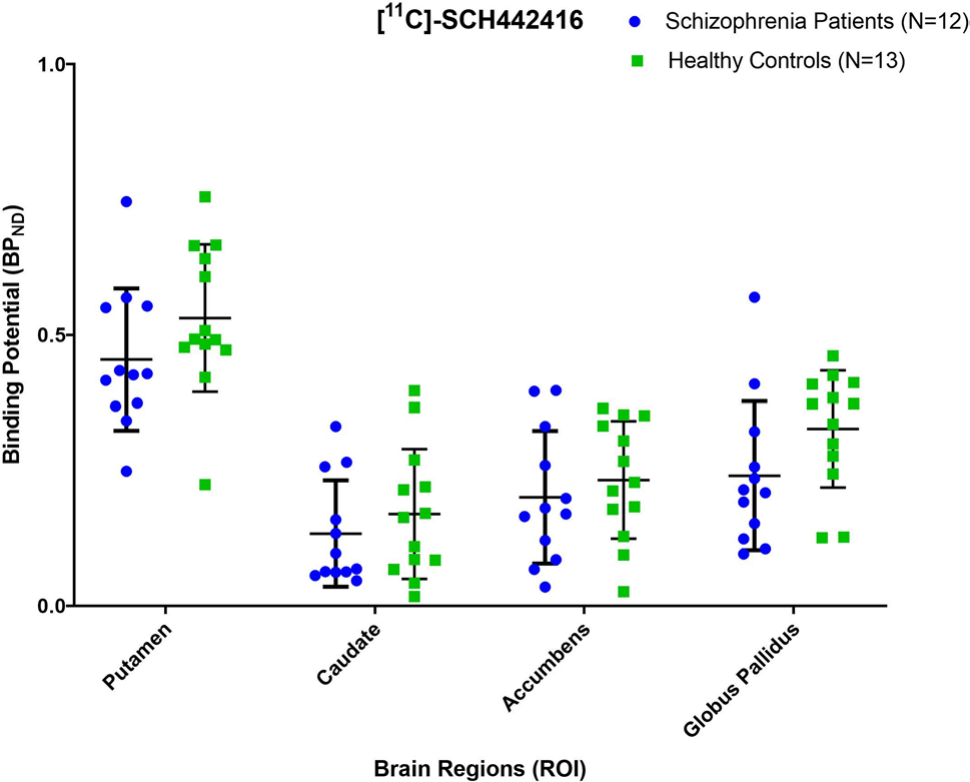
Scatterplot comparison of [11C]SCH442416 BP_ND_ between patients with schizophrenia and healthy volunteers

There were no differences in the injected dose, injected mass and specific activity between patients with schizophrenia and healthy controls (Table 1). Patients with schizophrenia showed no significant difference in [^11^C]SCH442416 BP_ND_ in the primary regions of interest ((caudate (*p* = 0.42, *t* = 1.42, df (23)) and putamen (*p* = 0.16, *t* = 0.82, df (23)) or in secondary regions of interest (globus pallidus (*p* = 0.09, *t* = 1.75 df (23)) and nucleus accumbens (*p* = 0.49, *t* = 0.69, df (23)) when compared to healthy controls (Table 2 Fig. 1). There was also no significant correlation between [^11^C]SCH442416 BP_ND_ and dose of antipsychotics (caudate (*r* =  − 0.33, *p* = 0.13), putamen (*r* = 0.27, *p* = 0.19), globus pallidus (*r* = 0.21 *p* = 0.28), nucleus accumbens (*r* = 0.09, *p* = 0.34)) or with severity of total psychotic symptoms ((caudate (*r* =  − 0.07,*p* = 0.82), putamen (*r* =  − 0.27, *p* = 0.39), globus pallidus (*r* =  − 0.12, *p* = 0.69), nucleus accumbens (*r* =  − 0.39, *p* = 0.20)). There was also no significant correlation between [^11^C]SCH442416 BP_ND_ and severity of positive ((caudate (*r* =  − 0.08, *p* = 0.80), putamen (*r* = 0.09, *p* = 0.76), globus pallidus (*r* = 0.10, *p* = 0.74), nucleus accumbens (*r* = 0.002, *p* = 0.99)), negative (caudate (*r* =  − 0.14, *p* = 0.65), putamen (*r* =  − 0.19, *p* = 0.55), globus pallidus (*r* =  − 0.19, *p* = 0.54), nucleus accumbens (*r* =  − 0.39, *p* = 0.20)) or general psychotic symptoms (caudate (*r* = 0.11, *p* = 0.72), putamen (*r* =  − 0.40, *p* = 0.18), globus pallidus (*r* =  − 0.14, *p* = 0.65), nucleus accumbens (*r* =  − 0.39, *p* = 0.19)). Finally, there was also no significant correlation between [^11^C]SCH442416 BP_ND_ and duration of illness ((caudate (*r* =  − 0.45, *p* = 0.14), putamen (*r* =  − 0.33, *p* = 0.29), globus pallidus (*r* =  − 0.45, *p* = 0.13), nucleus accumbens (*r* =  − 0.28, *p* = 0.37)).

**Table 2:** Comparison of [11C]SCH442416 BP_ND_ between patients with schizophrenia and healthy volunteers

	Patients (*n* = 12)		Healthy controls (*n* = 13)		Test statistic			
ROI	Mean	SD	Mean	SD	% difference	Effect Size	*F* (df)	*p*
Caudate	0.133	0.098	0.169	0.119	− 27%	0.33	1.42 (23)	0.42
Putamen	0.454	0.131	0.531	0.136	− 17%	0.57	0.82 (23)	0.16
Accumbens	0.200	0.122	0.232	0.108	− 16%	0.27	0.69 (23)	0.49
Globus Pallidus	0.240	0.137	0.326	0.107	− 36%	0.7	1.75 (23)	0.09

## Discussion

To our knowledge, this is the first study to assess the A2A receptor in vivo in patients with schizophrenia. We found no difference in striatal A2A binding potential between patients diagnosed with schizophrenia and healthy controls in any of the basal ganglia regions examined.

Several potential limitations affecting the interpretation of the results need to be considered. First, the challenges for quantifying A2AR with current in vivo ligands. The first measures of A2A receptors in healthy controls were made using xanthine tracers such as [^11^C]-TMSX, but these showed several problems such as a variable affinity at striatal and non-striatal A2AR, and hence non-xanthine tracers are preferred (Naganawa et al. 2007; Ishiwata et al. 2008; Tavares et al. 2013). [^11^C]SCH442416 is a non-xanthine tracer that has high affinity and selectivity for adenosine A2A receptors, showing higher striatal to cerebellum ratios than previous ligands and good signal-to-noise (Moresco et al. 2005; Brooks et al. 2010). However, the existence of a slow and irreversible component and considerable inter-subject variability are limitations for the use of this tracer. To overcome this limitation, we estimated BP_ND_ by fitting the SRTM to just the first 15 min of scan data, as this component of the signal is likely to best index the reversible binding to A2AR. There are blocking data using a drug selective for the A2A receptor that indicates fitting a SRTM model to the first 15-min scan data that identifies tracer binding that is specific to the A2A receptor (Gunn R. Personal Communication). Another approach has been to use spectral analysis to identify the different components of the signal (Subrata et al. 2010). The control BP_ND_ data from the study using the spectral analysis approach are lower than we obtained using our approach. However, it should be recognised that both our approach and spectral analysis approach for this tracer require further validation. Alternatively, future studies could make use of more recent radiotracers for imaging A2A with better kinetics properties, such as [^18^F]-MNI-444 (Barret et al. 2015) that were not available for human use when we started our study. Second, there has been suggestions that reduction in A2A receptor levels are only present in a subgroup of schizophrenia patients, particularly in those with DNA methylation of the gene coding for A2AR (ADORA2A). (Villar-Menéndez et al. 2014) Future PET imaging studies of the A2A receptor should be control for this potential molecular mechanism. Third, A2A receptors levels are dependent on the density of adenosine A2A receptors (A2ARs) but also on the degree of their functional heteromerization with dopamine D2 receptors (A2A-D2 heteromers). Changes in the degree of A2AR-D2R heteromerization could potentially counteract changes in A2A receptor levels. (Valle-León et al. 2021). Future studies should contribute to elucidate the close relationship between the expression of A2A and D2 receptor expression and A2A-D2 heteromers. It is also possible that other confounding factors may have impacted the results. Caffeine is known to exert its effect through a blockage of adenosine A2A receptors. As approximately 60% of patients with schizophrenia use coffee or caffeinated drinks (Gurpegui et al. 2004), it is possible that the use of this substance may have impact the results. However, we excluded patients and controls who consumed high levels of caffeinated beverages and subjects were asked to abstain from caffeinated products for > 24 h prior to PET scan. Given the plasma half-life of caffeine is approximately 5 h, this period of abstention allowed for wash-out of caffeine, reducing the risk that caffeine is affecting our findings (Brachtel and Richter 1992; Temple et al. 2017). Other recreational substances including cigarette smoking did not statistically differ from the control group. We have also detected moderate to large effect sizes both for the putamen (0.57) and the globus pallidum (0.7) although this did not reach a statistical significance. As the statistical significance is dependent on the sample size, the relatively small sample size may not have provided adequate power to detect significant group differences. Further investigation of potential effects in these regions would thus be useful. Finally, one potential limitation on generalizability is that we only included males. Although there are no studies reporting sex differences in A2A receptor expression, future studies are needed to test if our findings generalize to female patients as well.

Our results do not support the existing post-mortem imaging findings in schizophrenia. While Villar-Menéndez showed a reduction in A2AR binding, Kurmanji and Toru found an increase in approximately 35 to 55% in A2A receptor specific binding in the caudate and putamen of post-mortem brains, as measured by [3^H^]-CGS21680 (Kurumaji and Toru 1998; Villar-Menéndez et al. 2014). Also, Deckert et al., using this same tracer, have reported a 70% increase in Bmax in post-mortem striatal tissue of patients diagnosed with schizophrenia (Deckert et al. 2003). In Kurmanji and Toru study, the increase was seen both in patients that had received antipsychotics until immediately before death and others that were untreated for more than 40 days before death. One potential explanation for the discrepancy between these results and our findings is that the duration of illness and antipsychotic treatment in both studies far exceeds that of our present study. All antipsychotic drugs block D2 receptors, which, at least in rats exposed to chronic treatment with haloperidol and fluphenazine, leads to an upregulation of A2A (Parsons et al. 1995). Thus, the longer exposure to antipsychotics may have resulted in the increases in A2A receptors seen in the post-mortem studies. It is therefore theoretically possible that there is a reduction in A2A levels in schizophrenia, with antipsychotics gradually increasing the levels of this receptor, masking alterations in our in vivo study and leading to elevations in post-mortem studies where patients have been treated for many years. However, the study by Villar-Menéndez has assessed patients with chronic schizophrenia, with the majority of them hospitalised for more than 40 years, and no significant relationship between A2AR levels and antipsychotic exposure was observed (Villar-Menéndez et al. 2014). Moreover, we did not find a relationship between antipsychotic exposure and A2AR availability. Nevertheless, future studies in antipsychotic naive or antipsychotic-free patients are needed to definitively rule out the possibility that anitpsychotic exposure is masking alterations in A2AR in schizophrenia. Although our study does not suggest the A2AR as a primary pathophysiological target in schizophrenia, the close interaction of A2AR with D2 receptors does not exclude a potential role for the manipulation of this receptor as a potential pharmacological target in the treatment of this disorder Fig. 1.

In conclusion, we showed for the first time in vivo that there is no significant differences in A2A receptor expression between patients and healthy controls, not supporting the adenosine hypofunction hypothesis of schizophrenia.
